# Experimental Study on the Axial Compression Performance of an Underwater Concrete Pier Strengthened by Self-Stressed Anti-Washout Concrete and Segments

**DOI:** 10.3390/ma14216567

**Published:** 2021-11-01

**Authors:** Shaofeng Wu, Yijun Ge, Shaofei Jiang, Sheng Shen, Heng Zhang

**Affiliations:** 1 College of Civil Engineering, Yango University, Fuzhou 350015, China; shaofeng45@163.com (S.W.); geyijun@ygu.edu.cn (Y.G.); 2 Fuzhou Communication Construction Group Co., Ltd., Fuzhou 350008, China; 3 College of Civil Engineering, Fuzhou University, Fuzhou 350108, China; s_shen@126.com (S.S.); zhangheng57@126.com (H.Z.)

**Keywords:** self-stressed anti-washout concrete, segment assembly, undrained strengthening, axial compression test, mechanical properties

## Abstract

Compared with the conventional drainage strengthening techniques, the precast concrete segment assembly strengthening method (PCSAM) is regarded as a fast, more economical, and traffic-friendly underwater strengthening method for damaged bridge piers and piles, as the drainage procedure can be omitted. However, this method still has some disadvantages, such as strength loss of the filling material, debonding of the interface due to shrinkage of the filling material, poor connection effects, and poor durability of the segment sleeves. To solve these problems, the PCSAM is improved in this study by using self-stressed anti-washout concrete (SSAWC) as the filling material and by developing a lining concrete segment sleeve (LCSS) by referring to the design theory for shield lining segments. Six specimens are designed and prepared with consideration of the influential factors, such as the self-stress, thickness of the filled concrete, and concrete strength of the LCSS, then the monotonic axial compression test is carried out to investigate the improvements in the axial compression properties of the specimens. Accordingly, extended parametric analyses are performed based on the established numerical models. Finally, the calculation formula for the bearing capacity is proposed based on the analysis results. The results indicate that the SSAWC can provide initial confining compressive stress in the core region of the piers, in addition to increasing the bearing capacity and ductility of the specimens. The improved LCSS segment connection is more reliable and increases the strengthening efficiency. The influence of self-stress on the bearing capacity of the specimens is cubic and the influence of the filled concrete strength on the bearing capacity of the specimens is nonlinear. The calculation formula for predicting the bearing capacity of axially compressed columns possesses good applicability and can be used as a reference for practical engineering.

## 1. Introduction

Substructural components, such as piers and piles, are critical to the safety of bridge structures. Due to the low requirements of design codes decades ago, material degradation, and environmental erosion, the existing components generally suffer from delamination of the concrete covers, exposed ribs, and riverbed cutting. These defects are not easy to be detected because the substructure components are almost underwater throughout their life cycle, which limits the function of such bridges, even leading to collapse. Therefore, many conventional strengthening methods and techniques, such as the bonded steel plate method [1], enlarging section method [2], planting bar method [2], fiber-reinforced polymer (FRP) method [3,4], and prestressed strengthening technique [5], have been proposed and applied in practical engineering. Although these techniques have perfect design theories, mature construction techniques, and good strengthening effects, they are time-consuming, expensive, and traffic-disrupting because of the necessary construction of cofferdams, which are used for drainage before strengthening works. Consequently, increasing attention has been paid to undrained strengthening technology recently.

A number of undrained strengthening techniques have been developed and applied to practical engineering in the past decade, such as the jacket strengthening method [6], FRP underwater strengthening method [7,8], and precast concrete segment assembly method (PCSAM) [9]. These methods are economical, fast, and traffic-friendly compared with conventional methods, but also have some fatal deficiencies. For instance, the jacket strengthening method can greatly improve the durability of components but piers cannot be wrapped in fiber sleeves in deep water, which leads to strengthening failure. For the FRP underwater strengthening method, although the underwater strengthening effect is good, the empty gap generally occurs in the interface between the FRP and components because of the ineliminable water. As a result, this method tends to fail when the piers are submerged in water for a long time after strengthening. Additionally, the diving operation involves high costs, safety risks, and slow progress in deep water and under certain water pressure and flow rates. On the contrary, the PCSAM first uses precast concrete segments to wrap the components, then the segments are connected into the sleeve using wire ropes, and finally the interspace between the sleeve and the components is filled with the filling material to achieve undrained strengthening. The PCSAM can be used to strengthen the components in deep water. This approach has many advantages, including the simple construction method, reliable construction quality, short construction period, and low cost. However, there are still some problems with the PCSAM, such as the large prestress loss and poor durability of the wire ropes, poor accuracy and connection performance of the sleeves, large strength loss of the filling material, and uncertain bonding properties between the filled concrete and sleeves. As a consequence, there is an urgent need to solve the problems existing in the PCSAM and to develop a new strengthening method that can better serve in the reinforcement of damaged components.

Based on the PCSAM, this paper proposes a new undrained strengthening method named the improved precast concrete segments assembly method (IPCSAM), which takes advantage of three theoretical reinforcement techniques, namely the increasing section method, outer sleeve method, and prestressing method. The IPCSAM proposed in this paper not only uses self-stressed anti-washout underwater concrete (SSAWC) as the filling material, but also improves the connection of the segment sleeve, which is referred to as the shield lining segment. The SSAWC, as the filled concrete, can not only cut down the strength loss of the underwater concrete [10,11,12], but can also increase the bonding strength between the filled concrete and the sleeves [13]. On the one hand, due to the additional appropriate expansion agent added to the SSAWC compared with the AWC, the concrete becomes denser and the self-stress makes the bond between the sleeve and the filled concrete stronger. On the other hand, the shield lining segments have high precision in prefabrication [14], good connection effects [15], and good durability [16]. The lining concrete segment sleeve (LCSS) is developed according to the characteristics and theory of shield lining segments under the consideration of existing problems, while the prefabrication precision, durability, and joint connection strength of the segment are improved.

The axial compression performance of the strengthened columns is so significant that numerous studies have investigated this topic. Fakharifar [17] found that the bearing capacity and ductility of columns strengthened with steel sleeves could be increased by 20%. Wang [18] used a CFRP sleeve to strengthen a column, the bearing capacity of which was greatly improved. In [9], the peak load and displacement of the column strengthened by the PCSAM were increased by 28% and 20%, respectively. Seible [19] proposed a design method for strengthening columns with FRP sleeves, while Tang and Wu and Sun [20] proposed a simplified moment–curvature calculation model for the cross-section of the column strengthened by the PCSAM.

This paper attempts to investigate the strengthening effect of the IPCSAM and the axial compression performance of the strengthened columns. Firstly, the implementation strategy for the IPCSAM is introduced. Then, 6 specimens are prepared by taking account of influential factors, such as self-stress, the thickness of filled concrete, and the concrete strength of the LCSS, while the mechanical properties of the specimens are also studied under axial compression loading. Next, the extended parameter analyses are carried out via numerical simulations. Finally, based on the experimental results and extended parameter analyses, the calculation method for the bearing capacity is established for the components strengthened with the IPCSAM.

## 2. IPCSAM

### 2.1. SSAWC Preparation

The use of self-stressed anti-washout underwater concrete (SSAWC) is one of the key strategies for implementing the IPCSAM, because the filling material connects the components and segment sleeves and directly affects the mechanical properties of the strengthened components. Wu and Jiang [10] developed the SSAWC and achieved good performance. The compressive strength ratio in water compared to air for the SSAWC increased from 0.8 to 0.92, indicating that the strength loss of the filling material was significantly reduced. The restrained expansion ratio at 14 days for SSAWC could reach 0.027–0.053%, while the self-stress generated by expansion would be beneficial to increasing the bond strength [13]. Meanwhile, the experiments carried out by García-Calvo [12] showed that the underwater environment was more beneficial to the self-stress development of the SSAWC for piers and columns.

According to the mix ratio of the SSAWC shown in Table 1 and proposed by Wu [10], performance verification tests for the C30 SSAWC were carried out. The 42.5 *N* normal Portland cement from Fujian Cement Inc. (Fujian, China), a fiber-type anti-dispersion agent named SBTNDA, and the HME-III low-alkali concrete expansive agent produced by the Jiangsu Soubotte Company (Nanjing, China) were chosen. A polycarboxylate superplasticizer was employed in these tests based on experimental results from Khayat [21] and Oliveira [22]. Here, 5~20 mm of continuous-grade gravel was used. The density, crushed index, needle content, and sediment content of the gravel were 2.65 t/m^3^, 7.3%, 2%, and 0.2%, respectively. Good Fujian River sand was used, the fineness modulus, sediment content, and density of which were 2.89, 0.6%, and 2.64 t/m^3^, respectively. The test results showed that the C30 SSAWC (Table 2) had a higher elastic modulus and underwater strength compared with the AWC and the expansibility of the SSAWC could be easily observed.

### 2.2. LCSS Preparation

The use of a lining concrete segment sleeve (LCSS) is another key strategy for implementing the IPCSAM, as the sleeve can be used as formwork in the construction process and as a part of the strengthened piers to bear partial loads, in addition to being a limitation that makes the SSAWC produce self-stress. Huang [14] studied the precast technology used for the shield lining segments and found that the prefabrication precision could be improved by controlling the working procedures to achieve assembly error within 2 mm for the inner ring segments. In the experiments conducted by Liu [15], the failure of the shield tunnel linings was caused by failure of the joints, while the bearing capacity of the joints was improved using strengthening bolts. The study by Meng [16] showed that the durability of the shield lining segment could be improved by adding steel or propylene fibers or steel bars into the concrete. Therefore, the LCSS was designed and prepared with the characteristics and theory of shield lining segments.

**Figure 1:**
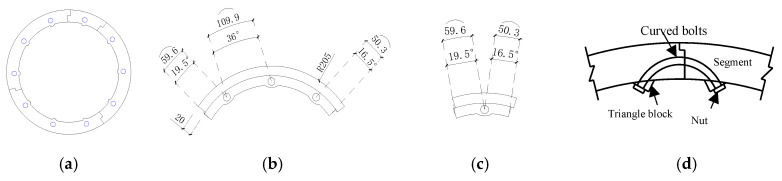
Structure and connection of the LCSS. (**a**) Segment ring; (**b**) Standard block; (**c**) Adjusting block; (**d**) Circumferential connection.

**Figure 2:**
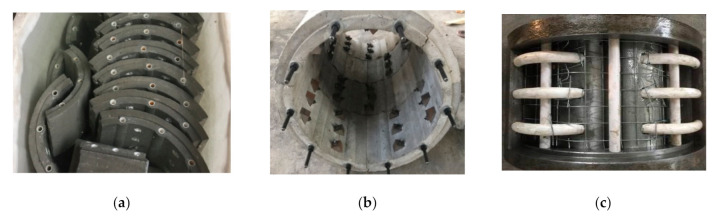
The LCSS and mold. (**a**) Segments; (**b**) Sleeve; (**c**) Mold of segments.

The LCSS design was divided into structural and connection designs. The structural design determined the preassembly form and segment dimensions. The LCSS was divided into two parts, namely the standard segments and adjusting segment; the sleeve ring consisted of three standard segments and one adjusting segment, as well as a setting rabbet between the segments. The dimensions of the segment were determined based on the self-stress, load, thickness of the concrete cover, diameter of the PVC pipe, and the construction technique. The structure of the LCSS is presented in Figure 1, and the segment dimensions of the specimens are designed as follows: inner diameter of 165 mm, outer diameter of 205 mm, thickness of 40 mm, height of 170 mm. The connection for the LCSS was designed in the same way as the connection for the shield lining segments. Specifically, for circumferential connection of the segments, the holes for fixing bolts were reserved through the pre-embedded PVC pipes and then the curved bolts and triangular pad were utilized to connect the segments. Regarding the longitudinal connection of the segment, the hole was also reserved first, then the long bolts and nuts were used to connect the segments. In this test, the diameter of the PVC pipe was 16 mm and the diameters of the circumferential and longitudinal bolts were 6 mm and 8 mm, respectively. Three circumferential connections were arranged along the longitudinal equal spacing of each segment ring, and 10 longitudinal connections were arranged at equal intervals along the circumference; the connections of the segments are shown in Figure 1. The prefabricated mold of the LCSS was designed and is shown in Figure 2. The size of the segments and location of the PVC pipe complied with the requirements found in [14].

## 3. Experimental Procedures

### 3.1. Experimental Design

The objective of this test was to investigate the influence of the SSAWC and LCSS on the performance of the specimens, as well as the impacts of parameters such as the segment concrete strength and filled concrete thickness on the bearing capacity of the strengthened specimens. Therefore, the experimental design was carried out according to the test objectives.

#### 3.1.1. Specimens Design

(1) Dimensions of the specimens: The strengthened specimens were composed of three parts: the lining concrete segment sleeve (LCSS), the unreinforced column, and the filled concrete (SSAWC). Referring to the specimen designs found in relevant studies [17,23], 1/5 model proportions were adopted to avoid difficulties in preparation and deviation of the test results due to the small size. The dimensions of the unreinforced columns were as follows: diameters of 250 mm and 200 mm, height of 750 mm.
(2) Representativeness and number of test specimens: The control variables of the test specimens selected in this paper were the diameter of the unreinforced column, axial compressive strength, thickness, self-stress, and axial compressive strength of the LCSS. In order to investigate the influence of the above variables and ensure the specimens were representative, the initial values of the control variables were determined by the code in [24], involving existing pier-strengthening technologies and the design parameters of ordinary piers. The diameter of the unreinforced column was 250 mm as a result of the 1/5 scale, the axial compressive strength of the filled concrete was 21.7 MPa, the thickness was 40 mm as a result of the 1/5 scale, and the axial compressive strength of the segment concrete was 20.9 MPa. Because it took a lot of time to prepare the reinforced test specimens, time and funds were limited when three specimens were prepared under the same parameter conditions. For the sake of speeding up the test process, the following strategy was adopted. According to reference [9], it was known that the errors for three specimens with the same parameters are generally within 10%. Therefore, only one specimen with the same parameters was prepared in this paper. In order to ensure the rationality and accuracy of the test data, the finite element model with the same parameters was established and the parametric analysis was performed. Based on the above principles, as well as the premise of each variable with a control group, a total of 6 test specimens were prepared in this paper, as shown in Table 3.
(3) Specimen grouping: The specimens were divided into three groups according to the objectives of this test. Table 3 shows the details. (1) The unreinforced group (UG) had two specimens with diameters of 200 mm and 250 mm respectively, which were used for comparison with the corresponding strengthened specimens to verify the strengthening effect. (2) The ordinary reinforced group (ORG) was a specimen strengthened with AWC, which was used for comparison with specimens in the other group to verify the influence of the SSAWC and the LCSS on the strengthening effect. (3) The self-stressed reinforced group (SRG) was composed of specimens filled with SSAWC, among which the concrete strength of the LCSS of specimen S1-SS was reduced to verify the influence of the strength of the sleeve concrete on the bearing capacity of the strengthened specimens, while the thickness of the filled concrete of specimen S2-S was increased to verify the effect of the thickness of the filled concrete on the bearing capacity of the strengthened specimens.

#### 3.1.2. Meter Placement Design

**Figure 3:**
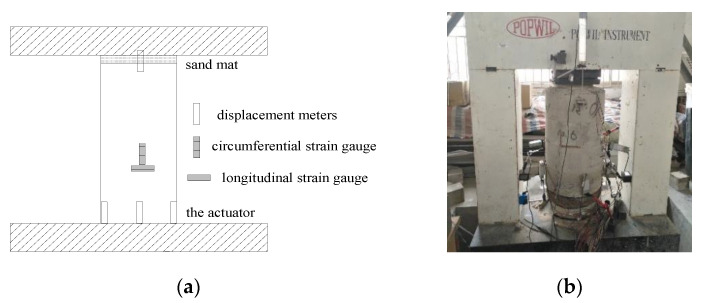
The placement of displacement meters and strain gages. (**a**) Design positions of meters; (**b**) Actual positions of meters.

**Figure 4:**
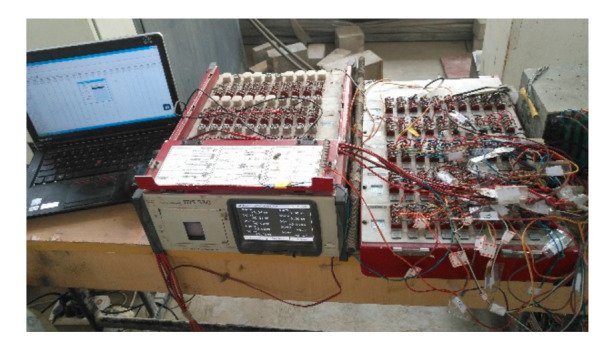
TDS-530 static data collector.

The positions of displacement meters and strain gauges are given in Figure 3. A YHD-50 displacement meter was fixed at the top of the specimen to record the deformation of the sand mat, which was used to eliminate the impact of the uneven loading plate. Four YHD-50 displacement meters were fixed on the actuator of the loading device at equal angle intervals to record the longitudinal displacement of the specimen. At the half height of the specimen, four 100 mm concrete strain gauges were fixed on the outer surface of the sleeve along the circumferential and longitudinal directions to record the circumferential and longitudinal strains of the sleeve, respectively. At the same time, at the half height of the self-stressed reinforced specimens, four 80 mm concrete strain gauges were fixed on the inner surface of the sleeve along the circumferential and longitudinal directions to record the circumferential and longitudinal strains of the filled concrete, respectively. Both the displacement and strain time histories were recorded using a TDS-530 static data collector with an acquisition frequency of 0.5 Hz, as shown in Figure 4.

#### 3.1.3. Loading Design

Based on the estimation of the bearing capacity of the specimens, a 300 t pressure loading instrument was used. Firstly, the pressure was loaded at 15 kN/min. Then, when the load exceeded 50% of the bearing capacity, the displacement was loaded at 0.06 mm/min until the failure of the specimen.

### 3.2. Specimens Preparation

#### 3.2.1. Materials and Properties

(1) Concrete: The materials, mix ratio, and properties of the SSAWC are shown in Section 2.1. The materials and mix ratio of the AWC were the same as those of the SSAWC, although the expansive agent was replaced by cement equivalently. The common concrete was composed of the same raw material as the SSAWC, while the two strength grades of segment concrete were C30 and C20, respectively, and the concrete strength grade of the unreinforced column was C30. The mix ratio of the AWC and common concrete are shown in Table 4 and the performance is shown in Table 5. The axial compressive strength of the concrete was taken as the concrete strength. For different production times, the concrete strength levels of the segments and unreinforced columns were slightly different.
(2) Steel: In order to improve the durability of the segment [16], the equivalent substitution method was adopted to replace the structural reinforcement with welded steel wire mesh with a diameter of 1.2 mm as the structural reinforcement for the segment. The properties of the welded wire mesh, circumferential bolts, and longitudinal bolts are shown in Table 5.

#### 3.2.2. Production Process

**Figure 5:**
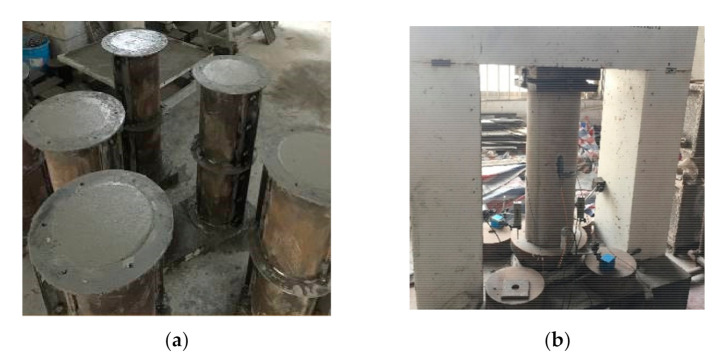
Unreinforced specimens. (**a**) Specimens pouring; (**b**) Completed specimen.

**Figure 6:**
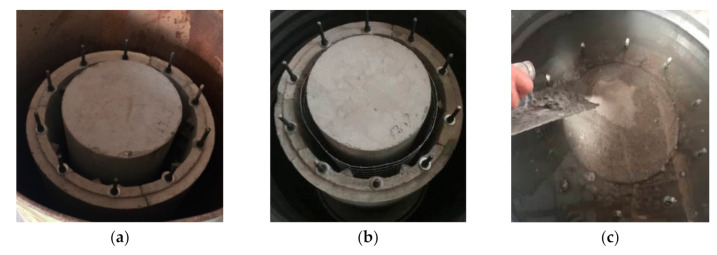
The production process for the strengthened specimens. (**a**) Preparing columns and LCSS; (**b**) Laying welded wire fabric; (**c**) Pouring SSAWC.

(1) Unreinforced specimens: As shown in Figure 5, unreinforced columns were made with a steel mold.
(2) Strengthened specimens: A bucket with a diameter of 600 mm and a height of 950 mm filled with water was used to simulate the underwater environment. The production procedure is shown in Figure 6 and was as follows: (**a**) prepare the LCSS and unreinforced column; (**b**) place the reinforcement mesh between the LCSS and column; (**c**) fill water into the bucket, pour the SSAWC and cure the concrete. It is noted that the concrete surface of the unreinforced columns should be chiseled manually before pouring concrete to increase the bond strength. In order to simulate the actual working state of the strengthened specimens and decrease the test errors caused by unbalanced loading, the areas with lengths measuring 35 mm were reserved without strengthening at the top and bottom of the specimens.

### 3.3. Failure Process and Modes

**Figure 7:**
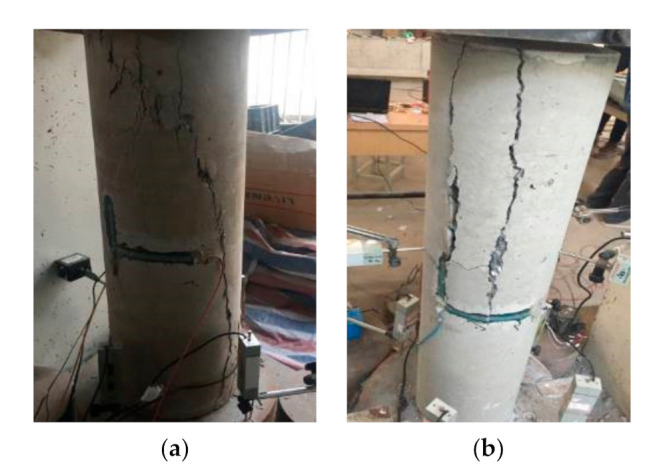
Failure of the unreinforced specimens. (**a**) P1 axial compression failure; (**b**) P2 axial compression failure.

**Figure 8:**
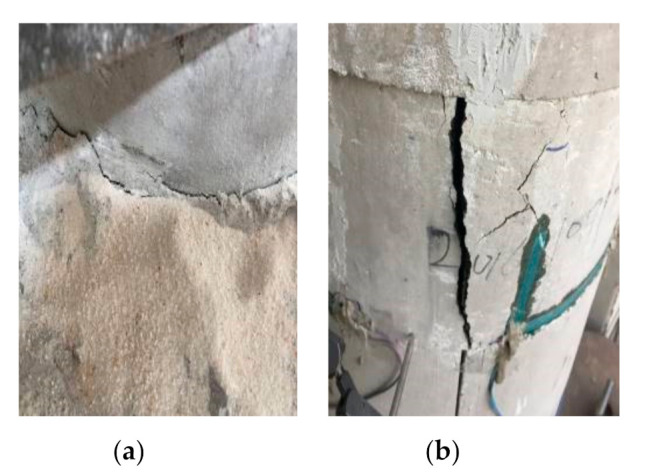
Failure of ordinary strengthened specimen. (**a**) Interface cracks; (**b**) LCSS fractures.

**Figure 9:**
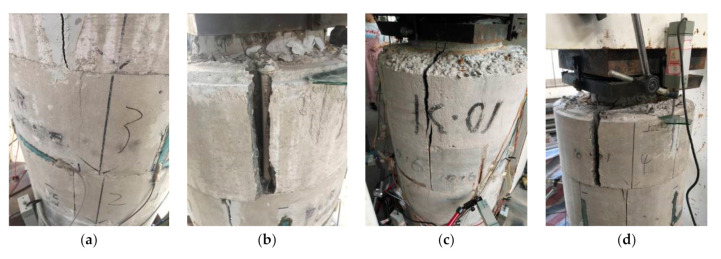
Failure diagram of self-stressed strengthened specimens. (**a**) Cracks on LCSS of specimen S1-S; (**b**) Fractures on LCSS of specimen S1-S; (**c**) Fractures on LCSS of specimen S1-SS; (**d**) Fractures on LCSS of specimen S2-S.

(1) Unreinforced specimens: As shown in Figure 7, the failure processes for P1 and P2 were similar. For specimen P1, the first crack in the concrete appeared when the load was close to 800 kN, which expanded with the loading. When the load was close to 1100 kN, some of the cracks were connected together to form a long and oblique crack. As a result, the bearing capacity of P1 reached the peak value. Then, the load dropped rapidly and specimen P1 reached failure. For specimen P2, the first crack in the concrete appeared when the load was close to 500 kN, which turned into a penetrating crack when the load was close to 800 kN. As a result, the bearing capacity of specimen P2 reached the peak.
(2) Ordinary strengthened specimen: Figure 8 shows the failure of specimen S1-A. Under the load of 1760 kN, the first concrete crack appeared at the interface between the filled concrete and the column. Then, with the increase in the load, the concrete crack gradually developed towards the segment sleeve and crisp sound could be heard, which was judged to be the fracture of the welded steel wire mesh. When the load was close to 2147 kN, several segments fractured and the bearing capacity of S1-A reached its peak, then the bearing capacity dropped until the failure of S1-A. The cracks were concentrated in the concrete cover of the longitudinal bolt, as well as the joint of the LCSS and the interface between the filled concrete and the column. It should be noted that all bolts remained unbroken.
(3) Self-stressed strengthened specimens: The failure process and modes of the three strengthened specimens were similar, as shown in Figure 9. When the load was close to 2000 kN, the first crack appeared in specimen S1-S, then the crack developed with the increases in load, making a cracking sound in the process. When the load reached 2600 kN, several segments broke and the bearing capacity of specimen S1-S reached the peak and stabilized for a period of time. Then, the bearing capacity dropped and the cracks appeared at the interface between the filled concrete and the column when the bearing capacity dropped to 2250 kN. Finally, as the load decreased continuously, multiple cracks penetrated and the specimen failed. When specimen S1-SS was loaded to 1730 kN, cracks of concrete appeared in the segment concrete. When the load was close to 1850 kN, interface cracks appeared and developed toward the segments. When the load was close to 2150 kN, the segments at the top of the specimen almost failed and the bearing capacity reached the peak, then bearing capacity continued to decrease with long penetrating cracks appearing in many places until the specimen failed. When specimen S2-S was loaded to 1500 kN, concrete cracks appeared in the segment. When it was loaded to 1920 kN, fractures occurred in the segment due to the penetration of the cracks and the bearing capacity reaching the peak. Then, the bearing capacity decreased with the appearance of long penetrating cracks and the specimen finally failed. In this group, the cracks were concentrated on the concrete cover of the longitudinal bolt and the segment joints and none of the bolts were broken. Through testing and calculation, the self-stress values of the reinforced specimens could be obtained, as shown in Table 6.

### 3.4. Load–Displacement Curves

**Figure 10:**
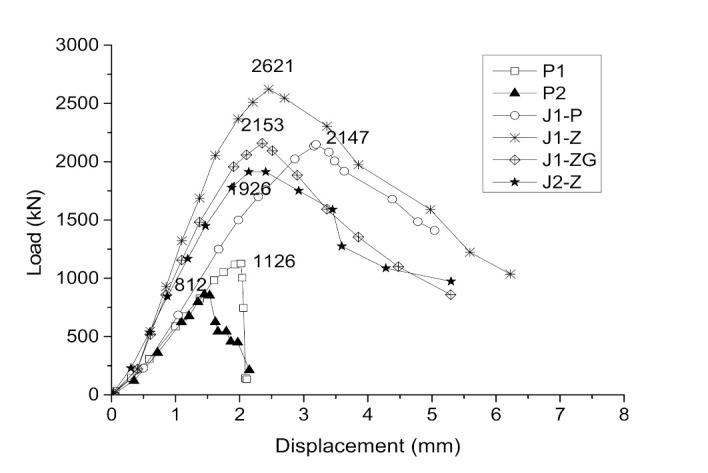
Load–displacement curves of the specimens.

Figure 10 shows the load–displacement curves of specimens. The curves of all the specimens consisted of three stages: elasticity, elastoplasticity, and failure. In these curves, the load–displacement relationship of the unreinforced column was consistent with the axial compression test of the plain concrete column [25]; that is, from the beginning to 80% of the peak load was the elastic stage, then the crack occurred at the beginning of the elastoplastic stage and the slope of the curve gradually decreased until the bearing capacity reached the peak, and finally the bearing capacity decreased rapidly until failure. It should be noted that the failure characteristic for specimen P1 was brittle failure. For the strengthened specimens, the load–displacement curves were similar. From the beginning to 80% of the peak load was the elastic stage. After this, the stiffness of the specimens gradually decreased with the development of the crack and the bearing capacity reached the peak when several segments broke. This process was referred to as the elastoplastic stage. Finally, the failure stage was reached. The bearing capacity of the specimen decreased until it failed and the descending speed was slower than that of the unreinforced column, showing the ductile failure characteristics of the strengthened specimens. The slopes of the load–displacement curves for the strengthened specimens were larger than those of the corresponding unreinforced columns, indicating that the stiffness increased after strengthening. In the same way, the slopes of the curves for the self-stressed strengthened specimens were larger than that of the ordinary strengthened specimen, indicating that self-stress enhances the stiffness of the strengthened specimens.

### 3.5. Load–Strain Curves

**Figure 11:**
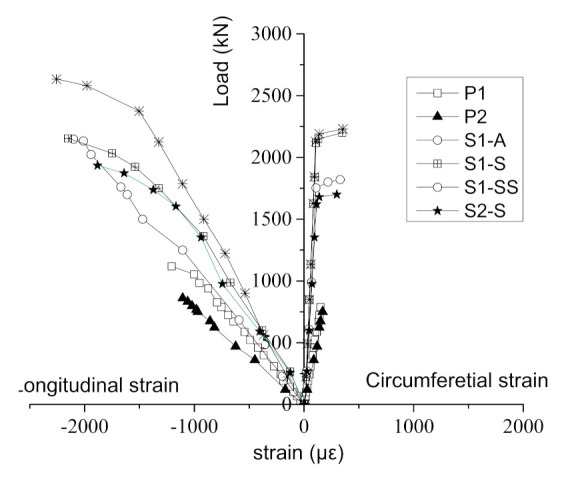
Load–strain curves of the specimens.

Figure 11 shows the load–strain curves of the specimens. For unreinforced columns, only the data before the peak load were analyzed, because the concrete strain was unstable and inaccurate after the peak load. Before the loads of P1 and P2 reached their peaks, the longitudinal and circumferential strains increased linearly. When it was close to the peak load, the longitudinal strain curve showed a downward bending trend with faster strain growth, which was similar to the axial compression in concrete column [9,25]. For the strengthened specimens, the load–strain curve was similar, whereby the longitudinal strains generally increased linearly and the downward bending tends appeared when it was close to the peak. The ultimate strains were much larger than for the unreinforced columns, which was in agreement with the conclusion found in [9,20]. The circumferential strains were small before the peak load and the strain increased obviously when the load approached the peak. This was due to the segments limiting the circumferential deformation of the specimens before the peak load, while the circumferential strains increased significantly after the failure of the segments.

### 3.6. Results and Discussion

Table 7 indicates the axial compression test data for the specimens. The results of the test were reasonable and were consistent with the related studies [9,10,11,12,20,25]. Hence, the correctness of the axial compression test on the specimens was verified. According to the objective of this test, the test results were compared to verify the reinforcement effect of the IPCSAM and to analyze the impacts of the reinforcement parameters on the axial compression performance of the strengthened specimens.

(1) The strengthening effect of the IPCSAM: The comparison of the test results showed that the peak loads of strengthened specimens S1-A, S1-S, S1-SS, and S2-S2 increased by 91%, 132%, 73%, and 137%, respectively, while the peak strains increased by 58%, 32%, 27%, and 61%, respectively. This meant the bearing capacity and ductility of the strengthened specimens had been significantly improved when compared with the unreinforced specimens, while the strengthening effect of the IPCSAM was remarkable.
(2) The influence of the LCSS on the strengthening effect: Since the existing relevant research results are very limited, the results from this paper were only compared with [9]. The comparison of specimen S1-A with the specimen YZ-SS strengthened by the PCSAM [9] showed that for S1-A, the ratio of the cross-sectional area before and after strengthening was A_1a_ = 2.69, the ratio of the bearing capacity before and after strengthening was B_1a_ = 1.91, and the strengthening efficiency was E_1p_= B_1a_/A_1a_= 0.71; for YZ-SS, the ratio of the cross-sectional area before and after strengthening was Ass = 1.96, the ratio of the bearing capacity before and after strengthening was B_ss_ = 1.28, and the strengthening efficiency was E_ss_ = B_ss_/A_ss_ = 0.65. In other words, the strengthening efficiency increased by 9% using the LCSS instead of the sleeve formed by wire ropes. At the same time, the peak strain of S1-A was also larger than that of YZ-SS, showing the better ductility of S1-A than that of YZ-SS.
(3) The influence of the SSAWC on the strengthening effect: The comparison between the two specimens S1-A and S1-S showed that the self-stress increased the bearing capacity by 41%. For S1-S, the ratio of the bearing capacity before and after strengthening was A_1s_ = 2.69, the ratio of the bearing capacity before and after strengthening was B_1z_ = 2.32, and the strengthening efficiency was E_1z_ = 0.86. In other words, the strengthening efficiency increased by 21% using the SSAWC as filled concrete. The reason was that the self-stress produced by SSAWC caused the column to be compressed in three directions, improving the bearing capacity and stiffness. At the same time, the first crack appeared on the interface between the filled concrete, the column of S1-A, and the segment of S1-S specimen. This indicated that self-stress could compensate for the shrinkage of the filled concrete and also contributed to the initial stress on the interface between the filled concrete and adjacent components, improving the bond strength as well as delaying the occurrence of interfacial cracks. Therefore, the SSAWC had significant effects on improving not only the bearing capacity and rigidity of the specimen but also the bond strength.
(4) The influence of the strength of the LCSS concrete on the strengthening effect: It was observed from the comparison of S1-S and S1-SS that the reduction of the LCSS concrete strength would directly affect the bearing capacity and peak strain of the strengthened specimen. This was because the LCSS was a component of the strengthened specimen; as the concrete strength of the LCSS was reduced, the load borne by the LCSS was also decreased. At the same time, the lower the concrete strength of the LCSS, the easier the sleeve failed. As such, the self-stress would be reduced due to the loss of the restriction provided by the sleeve, finally leading to the decreased bearing capacity.
(5) The influence of the thickness of the filled concrete on the strengthening effect: By comparing S1-S with S2-S, it was clear that the cross-sectional areas of these two strengthened specimens were the same, although the larger the thickness of filled concrete, the lower the bearing capacity of the specimen. The bearing capacity was borne by the filled concrete of the strengthened specimens, which complied with the code in [24], while the utilization factor of the filled concrete was 0.8 compared with the column; hence, for S2-S, the thickness of the filled concrete was larger than S1-S, so the bearing capacity was lower than S1-S.

## 4. Axial Compression Bearing Capacity

The axial compression bearing capacity of the strengthened pier was related to parameters such as the cross-sectional area and strength of the SSAWC, the cross-sectional area and concrete strength of the LCSS, and the self-stress value product produced by the SSAWC. Due to the complexity, long production time, and high cost of the specimens, only one strengthened specimen was prepared for each parameter. Referring to relevant research [9], the test models were supplemented by the finite element models while considering the influence of different parameters. On the basis of the results of the tests and extended analysis, the calculation formula for the bearing capacity was established.

### 4.1. Extended Parameter Analysis

#### 4.1.1. Numerical Model

Numerical simulations were performed using ABAQUS and the numerical model was simplified as follows:

(1) The material parameters of the concrete, bolts, and welded wire mesh were adopted according to the measured values, as shown in Table 2 and Table 5. The solid element was used to simulate for concrete and the plasticity damage model implemented in ABAQUS was used as the constitutive relationship of concrete in the elastoplastic stage. Damage factors of the damage plasticity model were calculated according to the code [26]. The bolts and welded wire mesh were simulated with the wire element and the double broken line model was used as the constitutive relation of steel;
(2) Referring to [9], the connection between segments was simulated by hard contact, meaning the contact surface was only under compression. The connections between bolts and the segment concrete were simulated using the embedded connection. According to the working characteristics of the interface and referring to the study by Zhao [9] and Zhou [27], the binding connection was used to simulate the connection between the filled concrete and its adjacent members and the Coulomb friction model was adopted;
(3) The unreinforced parts of the specimens were not simulated. The top and bottom surfaces of the specimens were simplified and coupled into one point. Constraints were set on each direction of the point obtained from the top coupling and on the point obtained from the bottom coupling, except in the Z direction;
(4) Displacement loading was adopted and the initial stress was used to simulate self-stress.

**Figure 12:**
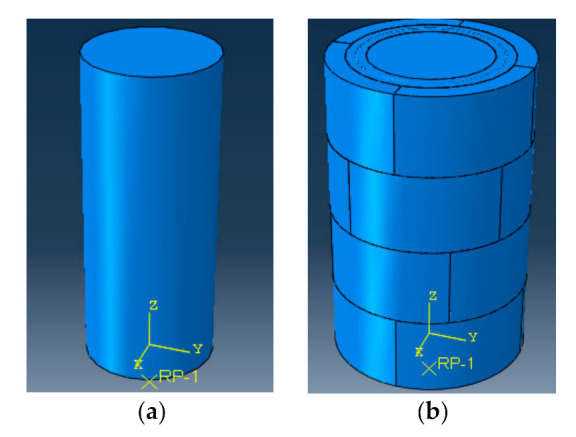
Finite element model. (**a**) Model of specimen P1; (**b**) Model of specimen S1-S.

**Figure 13:**
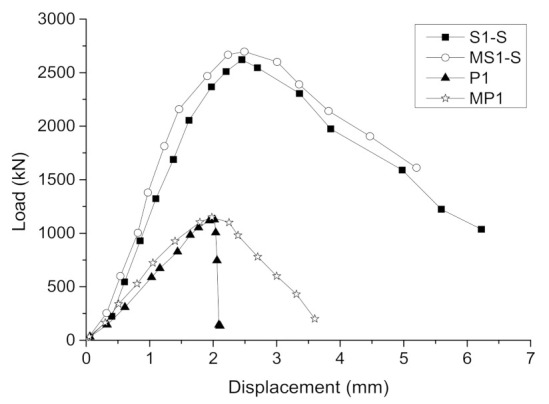
Load–displacement curves of specimens acquired using the numerical model and tests.

According to the above points, the finite element numerical model was established, as shown in Figure 12. Due to the difference between the simulated constitutive relationship and the actual constitutive relationship of the materials, as well as the influence of interface simulation and loading error on the test, the numerical model needed to be continuously adjusted according to the test results. The numerical model was adjusted with the peak load and load–displacement curve of actual specimens P1 and S1-S as the control index. By adjusting the constitutive relationship of the material, interface parameters, and loading system in the numerical model, the load–displacement curves of the specimen recorded in the tests and the numerical model were obtained, as shown in Figure 13. It can be seen that the load–displacement curves of specimens P1 and S1-S recorded in the tests were basically consistent with those recorded by the numerical model. The difference between peak loads was less than 5%, indicating that the numerical model was well in accordance with the specimens, meaning it could be used as the basic model for the extended parameter analyses.

#### 4.1.2. Parameter Extended Analysis

The influencing parameters of the axial compression bearing capacity for the strengthened specimens included the thickness and strength of the SSAWC, self-stress, and the thickness and concrete strength of the LCSS. The influence of the cross-sectional area of the filled concrete on the bearing capacity was considered in accordance with the code in [24]. Due to the limitations of the prefabricated mold used for the segments, the cross-sectional area of the LCSS basically remained invariable. Therefore, the influencing parameters were mainly self-stress, the strength of the SSAWC, and the concrete strength of the LCSS. Among them, the strength of filled concrete was set to three grades: C35, C40, C50; the longitudinal stress values were set to 0.8 MPa, 1.0 MPa, and 1.2 MPa; and the concrete strength of the LCSS was set to three grades: C35, C40, C50. As such, 9 extended numerical models were built to study the influence of these parameters, with Table 8 showing the specific parameter settings and peak loads.

(1) The influence of self-stress on the bearing capacity: Table 7 and Table 8 show that when the self-stress value was between 0 and 1.2 MPa, the peak load of the strengthened specimens increased with the increase in the self-stress. When the self-stress increased by 0.2 MPa, the bearing capacity increased by about 100 kN. However, as the self-stress value increased, the growth rate of the peak load decreased. The reason for this was that after the stress increased, the LCSS was easily damaged and the restraint effect on column was reduced, resulting in the decrease in the bearing capacity.
(2) The influence of the strength of the SSAWC on bearing capacity: It can be seen from Table 7 and Table 8 that the peak load of the strengthened specimens increased with the increase in strength of the filled concrete. When the concrete strength increased by 1 MPa, the bearing capacity of the strengthened specimens increased by 35 to 45 kN. However, the higher the strength of the filled concrete, the lower the growth rate of the bearing capacity of the strengthened specimens. This was because after the strength of the filled concrete increased, the LCSS failed first, resulting in a reduction of the increase in the bearing capacity.
(3) The influence of the concrete strength of the LCSS on the bearing capacity: Table 7 and Table 8 show that the peak load of the specimens increased with the increase in concrete strength of the segment. When the concrete strength of the LCSS increased by 1 MPa, the bearing capacity of the strengthened increased by 35 to 60 kN. However, the higher the strength of the segment concrete, the smaller the growth rate of the peak load. The reason was similar to that of the strength of the filled concrete.

### 4.2. Bearing Capacity Calculation Model

According to the results of the tests and extended numerical analysis, the calculation method used for the axial compression bearing capacity of concrete members strengthened involving the enlarging section method found in [24], and Xu’s study on the axial compression bearing capacity of self-stressed, concrete-filled steel tube columns [28], the calculation model for the axial compression bearing capacity of specimens strengthened by IPCSAM was established. In light of the structure and working characteristics of the components strengthened by the IPCSAM, the following assumptions were made on the bearing capacity calculation model of the IPCSAM reinforcement members:

(1) The bearing capacity of the strengthened specimens was composed of three parts: the bearing capacity of the unreinforced column and the filled concrete, the bearing capacity improved by the confinement provided by the sleeve and the filled concrete, and the bearing capacity improved by self-stress;
(2) The bearing capacity of the column and the filled concrete was calculated according to the calculation method of the code in [24]. In [24], the bearing capacity of strengthened components was equal to the axial compression bearing capacity of the column plus 80% of the axial compression bearing capacity of the filled concrete. In this test, the larger the filled concrete, the greater the error, so the utilization coefficient of the filled concrete needed to be adjusted. The contribution of the LCSS on the bearing capacity of the specimens was neglected to simplify the calculation;
(3) According to the calculation method in [29], the improved bearing capacity of the sleeve and filled concrete constraints was calculated. As a result, the confinement coefficient was used to consider the improvement of the bearing capacity of the axial compression;
(4) According to [28], the calculation method used to improve the bearing capacity through self-stress and the relationship between the improved bearing capacity and the self-stress level (the ratio of the self-stress value to the strength of the SSAWC) involved cubic equations. According to this method, the tensile stress generated by the self-stress shall be less than the tensile bearing capacity of the sleeve concrete, so the self-stress shall not be greater than 2 MPa;
(5) According to the design data for previous bridges, the concrete strength grade of the substructure mostly did not exceed C30, so it was assumed that the concrete strength grade of the columns was not greater than C30. At the same time, the substructures constructed years ago had low reinforcement ratios and there were issues related to bar exposure and corrosion. Therefore, the reinforcement of the columns was ignored.

Based on assumption (1), the calculation formula for the axial compression bearing capacity of the strengthened specimens (*N*) was proposed as follows:

(1) $$N=N_{0}+N_{s}+N_{p}$$

where *N*_0_, *N_s_*, and *N_p_* are the bearing capacity of the column and the filled concrete, the bearing capacity improved by the confinement, and the bearing capacity improved by self-stress, respectively.

Based on assumption (2), the calculation formula for the bearing capacity of the unreinforced column and the filled concrete (*N*_0_) was proposed as follows:

(2) $$N_{0}=0.9\varphi \left[f_{co}A_{co}+f_{yo}^{\prime }A_{so}^{\prime }+\alpha (f_{sc}A_{sc}+f_{y}^{\prime }A_{s}^{\prime })\right]$$

where *φ* is the stability coefficient of members, taking the value according to code [26]; *f_c__o_* and *f_sc_* are the strength of the column concrete and filled concrete, respectively; *A_c__o_* and *A_sc_* are the cross-sectional areas of the column and filled concrete, respectively; *f_y__o_′* and *f_y_*′ are the strength values of steel bars in the column and filled concrete, respectively; *A_y__o_*′ and *A_y_*′ are the cross-sectional areas of steel bars in column and filled concrete, respectively; *α* is the utilization coefficient of the filled concrete.

According to [24] and Huang’s study [30], the value of α should be determined by the interface bonding between the column and filled concrete. Assuming that the influencing factors of the interface bonding are the roughness, concrete strength of the column, filled concrete strength, and the usage of an interface agent, the calculation formula of *α* is as follows:

(3) $$\alpha =A(0.15+0.025\Delta )\ln f_{c}\zeta_{s}$$

where *A* is the adjustment coefficient considering the preloading of concrete in the filled concrete, which is an undetermined constant; Δ is the roughness of the interface when Δ ≥ 2.5, taking Δ = 5 mm; *f_sc_* is the average strength of the column concrete and filled concrete; *ζ_s_* is the influence coefficient of the interfacial agent, which is an undetermined coefficient.

Based on assumption (3), the calculation formula for the bearing capacity improved by the confinement (*N*_0_) was proposed as follows:

(4) $$N_{s}=\beta \theta N_{0}$$

(5) $$\beta =B{\left(\frac{f_{l}}{f_{co}}\right)}^{0.7}$$

(6) $$\theta =\frac{A_{gc}f_{gt}+A_{gy}f_{gy}+A_{sc}f_{st}+A_{s}^{\prime }f_{y}^{\prime }}{A_{co}f_{co}}$$

where *θ* is the confinement coefficient when *θ* ≥ 0.25, taking *θ* = 0.25; *β* is the adjustment coefficient related to the concrete strength of the SSAWC, the LCSS, and the column; *B* is an undetermined constant; *f_st_*, *f_gt_*, and *f_gy_* are the tensile strengths of the SSAWC, the LCSS concrete, and steel bars reinforcement, respectively; *A_gc_* and *A_gy_* are the cross-sectional areas of the sleeve concrete and steel bars, respectively; the value of *f_l_* takes the minimum of *f_sc_* and *f_gc_*.

Based on assumption (4), the calculation formula for the bearing capacity improvement caused by self-stress (*N*_0_) was proposed as follows:

(7) $$N_{p}=\gamma N_{0}$$

(8) $$\gamma =C\eta^{3}+D\eta^{2}+E\eta$$

where, *γ* is the improvement coefficient; *η* is the self-stress level, which is the ratio of the longitudinal self-stress value to the SSAWC strength; *C, D,* and *E* are coefficients of cubic equations to be solved.

To sum up, the calculation formula of axial the compression bearing capacity of the components strengthened by the IPCSAM is as follows:

(9) $$N=0.9\varphi \left[f_{co}A_{co}+f_{yo}^{\prime }A_{so}^{\prime }+\alpha (f_{sc}A_{sc}+f_{y}^{\prime }A_{s}^{\prime })\right]\left(1+\beta \theta +\gamma \right)$$

### 4.3. Determination of Parameters

(1) The *φ* value: According to the code in [26], when *l*_0_/*d* ≤ 7, *φ* = 1 is used for the specimens.
(2) The *α* value: The values of A and *ζ_s_* can be determined by regression fitting the data from Table 7 and Table 8. Then, the relationship between *α* and Δ is as follows:

(10) $$\alpha =0.75(0.15+0.025\Delta )\ln f_{c}\zeta_{s}$$

The value of *ζ_s_* is 1 when no interface agent is used. When the filled concrete is SSAWC, the value of *ζ_s_* is 1.07.

(3) The *β* value: The values of B can be determined by regression fitting the data from Table 7 and Table 8. Then, the relationship between *β* and *f_l_*, *f_co_* is as follows:

(11) $$\beta =2.698{\left(\frac{f_{l}}{f_{co}}\right)}^{0.7}$$

(4) The *γ* value. The values of C, D, and E can be determined by regression fitting the data from Table 7 and Table 8. Then, the relationship between *γ* and *η* is as follows:

(12) $$\gamma =6.683\eta^{3}-58.275\eta^{2}+9.556\eta$$

For the derivative of Formula (12), the self-stress level corresponding to the *γ* maximum value can be obtained; that is, the optimum self-stress level of the strengthened component. The optimum self-stress level of the specimen strengthened by the IPCSAM is 0.0875.

### 4.4. Formula Verification

In this paper, the data from the tests and extended parameter analyses, as well as the data used for comparison with [9] and [20], were used to verify Formula (9). The verification results are shown in Table 9, whereby the symbol YZSS(YZFS) represents the specimens strengthened by the PCSAM corresponding to [9], while the symbol ZMR(ZRW) represents the specimens from Tang’s test corresponding to [20]. It can be observed in the table that the difference between the bearing capacity calculated by Formula (9) and the bearing capacity of the tested and simulated specimens is less than 13%, which shows good applicability.

## 5. Conclusions

This study sets out to improve the PCSAM with the SSAWC and the LCSS. Initially, the strengthening effects of the above two improvement measures on the bearing capacity of the strengthened specimens were verified using an axial compression test. Then, the extended parameter analyses were performed by establishing the numerical models. Finally, the calculation formula for the axial compression bearing capacity of the strengthened specimens was established. The following conclusions were made:

(1) The IPCSAM has good strengthening effects on underwater pier strengthening structures without causing undraining. Compared with the unreinforced column, the axial compression bearing capacity and peak strain of specimens strengthened by the IPCSAM increased by 90–130% and 30–60%, respectively. This indicates that the bearing capacity and ductility of the strengthened specimens were considerably improved;
(2) As the filling material, the SSAWC can reduce losses in underwater strength in concrete and can produce expansion and self-stress. The self-stress not only improves the bond strength between the filled concrete and the adjacent member, but also provides a preloading effect on the column, which can improve the strength and ultimate strain of the concrete. Compared with the specimen strengthened by AWC, the bearing capacity of the specimen strengthened by the SSAWC increased by 18% and the strengthening efficiency increased from 0.71 to 0.86;
(3) The LCSS improvement caused by the shield lining showed reliable connection performance, which remained in good condition when the specimen was broken. In the test, the LCSS showed good integrity and confinement, which could increase the bearing capacity of a specimen. Compared with the PCSAM, the strengthening efficiency of the specimen strengthened by the IPCSAM increased from 0.65 to 0.71;
(4) The strength of the filled concrete, the strength of the LCSS concrete, and the self-stress were the key parameters affecting the bearing capacity of the specimens. With the increase in filled concrete strength, the LCSS strength, self-stress, and bearing capacity increased accordingly;
(5) The calculation formula for the bearing capacity of the strengthened components has been proposed. The findings from this study show that the formula has good applicability and provides a theoretical design basis for the application of the IPCSAM.

In addition, preparing the specimens requires a large number of steel molds and careful concrete curing, which takes a lot of time. Considering the time needed and the restrictions on research and development funds, only a small number of specimens were tested and a numerical simulation was conducted. As a consequence, the above-mentioned conclusions and remarks are only validated by limited experimental and simulation results. More experimental data and numerical simulations are needed to validate the proposed formulas in the future.

## Figures and Tables

**Table 1:** The mix ratio of the C30 SSAWC (kg/m^3^).

Cement	Expansive Agent	Anti-Dispersion Agent	Sand	Stone	Water-Reducing Agent	Water
433.3	48.14	12.03	692	995	4.81	207

**Table 2:** Properties of the C30 SSAWC.

Slump (mm)	Slump Flow (mm)	Restrained Expansion Rate at 14 Days (%)	pH Value	Elastic Modulus (GPa)	Underwater Strength at 28 Days (MPa)	Strength at 28 Days (MPa)	Strength Ratio
240	435	0.040	10.9	30.2	32.4	38.8	0.835

**Table 3:** Parameters and grouping of specimens.

Specimen	Group	D × H *^1^ (mm)	ACSC *^2^ of Unreinforced Specimen (MPa)	Filled Concrete			ACSC of LCSS (MPa)
				ACSC (MPa)	Thickness (mm)	Self-Stress (MPa)	
P1	UG	250 × 750	20.1	21.7	0	0	20.9
P2		200 × 750	20.1	21.7	0	0	20.9
S1-A	ORG	250 × 750	20.1	19.8	40	0	20.9
S1-S	SRG	250 × 750	20.1	21.7	40	1	20.9
S1-SS		250 × 750	20.1	21.7	40	0.8	13.6
S2-S		200 × 750	20.1	21.7	65	0.6	20.9

Note: *^1^: D×H = the diameter and height of the unreinforced specimen. *^2^: ACSC = axial compressive strength of concrete.

**Table 4:** The mix ratio of the concrete (kg/m^3^).

Strength Grade	Cement	Expansive Agent	Anti-Dispersion Agent	Sand	Stone	Water-Reducing Agent	Water
C30 (AWC)	481.4	0	12.03	692	995	4.81	207
C30	405	0	0	661	1174	4.05	175
C20	321	0	0	758	1138	0	183

**Table 5:** Performance of the materials.

Material	Elastic Modulus of Concrete (GPa)	ACSC (MPa)	Axial Tensile/Yield Strength of Steel (MPa)	Poisson’s Ration	Area (mm^2^)
C30 concrete in column	30.0	20.1	2.01	0.2	
C30 AWC	30.7	21.2	2.10	0.2	
C30 concrete in segments	30.3	20.9	2.06	0.2	
C20 concrete in segments	26.1	13.6	1.58	0.2	
Welded wire mesh	180		210	0.3	1.13
Circumferential/longitudinal bolts	206		640	0.3	28.26/50.24

**Table 6:** Results of test.

Specimen	Circumferential Strain (με)	Longitudinal Strain (με)	Circumferential Stress (MPa)	Longitudinal Stress (MPa)
S1-S	14	62	0.20	0.99
S1-SS	11	52	0.16	0.83
S2-S	11	39	0.14	0.63

**Table 7:** Results of the axial compression test.

Specimen	Peak Load (kN)	Peak Displacement (mm)	Peak Strain	Peak Load Increased Ratio	Peak Strain Increased Ratio
P1	1126	1.97	0.0026	-	-
P2	812	1.45	0.0016		
S1-A	2147	3.15	0.0042	91%	58%
S1-S	2621	2.63	0.0035	132%	32%
S1-SS	2153	2.48	0.0033	73%	27%
S2-S	1926	2.33	0.0031	137%	61%

**Table 8:** Material parameters of the extended models.

Name	Filled Concrete Strength (MPa)	Longitudinal Stress (MPa)	Segment Concrete Strength (MPa)	Peak Load (kN)
M-1	21.7	0.8	20.9	2578
M-2	21.7	1	20.9	2695
M-3	21.7	1.2	20.9	2802
M-4	21.7	1	23.4	2810
M-5	21.7	1	26.8	2876
M-6	21.7	1	32.4	2992
M-7	23.4	1	20.9	2771
M-8	26.8	1	20.9	2859
M-9	32.4	1	20.9	2955

**Table 9:** Verification results of the fitting formula.

Name	BCT(S) *^1^ (kN)	ACSC of Column *f*_co_ (MPa)	Filled Concrete Strength *f*_sc_ (MPa)	Concrete Strength of LCSS *f*_gc_ (MPa)	Self-Stress *q* (MPa)	Confinement Coefficient *θ*	Self-Stress Level *η*	BCC *^2^ (kN)	Difference
S1-A	2147	20.1	19.8	20.9	0	0.176	0	2035	−5.20%
S1-S	2621	20.1	21.7	20.9	0.99	0.177	0.046	2565	−2.12%
S1-SS	2153	20.1	21.7	13.4	0.83	0.147	0.038	2243	4.19%
S2-S	1926	20.1	21.7	20.9	0.63	0.250	0.031	2116	9.89%
M-1	2578	20.1	21.7	20.9	0.8	0.177	0.037	2507	−2.77%
M-2	2695	20.1	21.7	20.9	1	0.177	0.046	2568	−4.71%
M-3	2802	20.1	21.7	20.9	1	0.177	0.055	2615	−6.67%
M-4	2810	20.1	21.7	23.4	1	0.182	0.046	2648	−5.78%
M-5	2876	20.1	21.7	26.8	1	0.192	0.046	2769	−3.71%
M-6	2992	20.1	21.7	32.4	1	0.204	0.046	2960	−1.06%
M-7	2771	20.1	23.4	20.9	1	0.180	0.043	2633	−4.99%
M-8	2859	20.1	26.8	20.9	1	0.187	0.037	2774	−2.96%
M-9	2955	20.1	32.4	20.9	1	0.196	0.031	3004	1.66%
YZSS1 *^3^	3578	39.8	17.9	44.2	0	0.075	0	3470	−3.03%
YZSS2 *^3^	3220	39.8	17.9	44.2	0	0.075	0	3470	7.76%
YZSS3 *^3^	3984	39.8	17.9	44.2	0	0.075	0	3470	−12.90%
YZFS1 *^3^	4640	39.8	17.9	44.2	0	0.125	0	4060	−12.51%
YZFS3 *^3^	4524	39.8	17.9	44.2	0	0.125	0	4060	−10.26%
ZMW1 *^4^	1120	20.1	32.8	0	0	0.155	0	991	5.87%
ZMW2 *^4^	936	20.1	32.8	0	0	0.155	0	991	−11.52%
ZRW1 *^4^	1027	20.1	45.5	0	0	0.128	0	1153	12.25%
ZRW2 *^4^	1071	20.1	45.5	0	0	0.128	0	1153	7.66%
ZRW3 *^4^	1113	20.1	45.5	0	0	0.128	0	1153	3.59%

*^1^: BCT(S) = Bearing capacity of specimens tested (or simulated). *^2^: BCC = Bearing capacity calculated by Formula (4). *^3^: The symbol YZSS(YZFS) represents the data from [9]. *^4^: The symbol of ZMW(ZRW) represents the data from [20].
